# Survival From Pediatric Out-of-Hospital Cardiac Arrest During Nights and Weekends: An Updated Japanese Registry-Based Study

**DOI:** 10.1016/j.jacasi.2022.01.005

**Published:** 2022-05-10

**Authors:** Tatsuma Fukuda, Naoko Ohashi-Fukuda, Hiroshi Sekiguchi, Ryota Inokuchi, Ichiro Kukita

**Affiliations:** a Department of Emergency and Critical Care Medicine, Toranomon Hospital, Tokyo, Japan; b Department of Emergency and Critical Care Medicine, Graduate School of Medicine, University of the Ryukyus, Okinawa, Japan; c Department of Acute Medicine, Graduate School of Medicine, The University of Tokyo, Tokyo, Japan; d Department of Health Services Research, University of Tsukuba, Ibaraki, Japan

**Keywords:** adolescent, cardiopulmonary resuscitation, child, off-duty hours, out-of-hospital cardiac arrest, work style reform, ALS, advanced life support, CPR, cardiopulmonary resuscitation, EMS, emergency medical service, FDMA, Fire and Disaster Management Agency, IHCA, in-hospital cardiac arrest, OHCA, out-of-hospital cardiac arrest

## Abstract

**Background:**

Disparities in survival after pediatric out-of-hospital cardiac arrest (OHCA) between on-duty hours and off-duty hours have previously been reported. However, little is known about whether these disparities have remained in recent years.

**Objectives:**

This study aimed to examine the association of outcomes after pediatric OHCA with time of day and day of week.

**Methods:**

This observational study analyzed the Japanese government-led nationwide population-based registry data of OHCA patients. Pediatric (<18 years) patients who experienced OHCA between 2012 and 2017 were included. A multivariable logistic regression model was used to examine the association of both time of day (day/evening vs night) and day of week (weekday vs weekend) with outcomes after OHCA. The primary outcome was 1-month survival.

**Results:**

A total of 7,106 patients (mean age, 5.7 ± 6.5 years; 60.9% male) were included. 1,897 events (26.7%) occurred during night hours, and 2,096 events (29.5%) occurred on weekends. Overall, 1,192 (16.8%) survived 1 month after OHCA. After adjusting for potential confounders, 1-month survival during day/evening (1,047/5,209 [20.1%]) was significantly higher than that at night (145/1,897 [7.6%]) (adjusted odds ratio: 2.31 [95% CI: 1.87–2.86]), whereas there was no significant difference in 1-month survival between weekdays (845/5,010 [16.9%]) and weekends (347/2,096 [16.6%]) (adjusted odds ratio: 1.04 [95% CI: 0.88–1.23]).

**Conclusions:**

One-month survival after pediatric OHCA remained significantly lower during night than during day/evening, although disparities in 1-month survival between weekdays and weekends have been eliminated over time. Further studies are warranted to investigate the mechanisms underlying decreased survival at night.

Out-of-hospital cardiac arrest (OHCA), a leading cause of mortality, is a major public health concern worldwide.^1^, ^2^, ^3^, ^4^, ^5^ In the United States, an estimated 155,000 to 350,000 cases of OHCA occur annually, and the survival rates of OHCA are 9% to 10%.^2^^,^^6^, ^7^, ^8^ Pediatric cases account for 2% to 6% of all OHCA cases, and their survival rates are 6% to 13%.^6^^,^^9^, ^10^, ^11^ In Japan, approximately 130,000 cases of OHCA occur annually, and <10% survive.^12^, ^13^, ^14^ The survival rate from pediatric OHCA, which accounts for approximately 1% of the total patients with OHCA, remains low (17%–19%),^12^^,^^15^^,^^16^ although it is higher than that of adult cases of OHCA.

To improve treatment outcomes, Japan’s health policy has promoted the establishment of more emergency and critical care centers that can provide advanced and highly specialized care for critically ill and injured pediatric patients 24 hours a day, 365 days a year (Supplemental Table 1).^12^^,^^17^

Although a previous study in Japan demonstrated worse survival for pediatric OHCA (who were treated from 2005 to 2011) during nights or weekends compared with days/evenings or weekdays,^16^ much less is known about how survival differences between off-duty hours (ie, nights and weekends) and on-duty hours (ie, days/evenings and weekdays) change over time through the increase in emergency and critical care centers. This information is important for evaluating the effectiveness of Japan’s health policy and identifying opportunities for quality improvement in pediatric resuscitation.

To address this knowledge gap, we evaluated survival rates for pediatric cases of OHCA (who were treated from 2012 to 2017) by time of day and day of week. We hypothesized that survival rates from pediatric OHCA presenting during off-duty hours would be similar to those during on-duty hours in recent years.

## Methods

### Study design and data source

This study was a registry-based analysis of pediatric OHCA patients in Japan. The All-Japan Utstein Registry is a government-led nationwide population-based registry of OHCA patients managed by the Fire and Disaster Management Agency (FDMA). OHCA related terminology is followed by the Utstein definitions.^1^^,^^12^^,^^18^ As previous studies have described in detail,^13^, ^14^, ^15^, ^16^ trained emergency medical service (EMS) personnel prospectively collected data on all OHCA patients who were transported to an emergency hospital by using Utstein-style uniform reporting.^18^^,^^19^ During the study period, almost all OHCA patients in Japan were included in this registry regardless of whether they had do not resuscitate (DNR) orders, because EMS personnel in Japan are not allowed to terminate out-of-hospital resuscitation except in specific situations (eg, decapitation, rigor mortis, livor mortis, or decomposition).

The FDMA collected data from 3 sources (ie, 1-1-9 dispatch centers, fire stations, and receiving hospitals) and integrated them into the All-Japan Utstein Registry system on the FDMA database server. The integrity, accuracy, and completeness of the data were ensured through logical internal checks with standardized software and FDMA certification.

### Study setting and participants

Japan comprises an area of approximately 378,000 km^2^ and a population of approximately 126 million, including approximately 20 million children (aged <18 years). Japan has a nationally uniform EMS system with universal health coverage.^20^^,^^21^ The emergency number 1-1-9 is free for anyone who needs an ambulance anytime and anywhere. Ambulance teams are provided by municipal governments through local fire departments. There were approximately 730 fire departments with dispatch centers in Japan during the study period.^12^ Although physician-staffed ambulances are not generally available, most ambulances include at least 1 emergency life-saving technician, a highly trained EMS personnel, who can perform some part of advanced life support (ALS) procedures. EMS personnel in Japan perform cardiopulmonary resuscitation (CPR) according to the Japanese CPR guidelines, which conforms with the International Liaison Committee on Resuscitation (ILCOR) Consensus on Science with Treatment Recommendations (CoSTR), although EMS personnel have different authorities depending on their completed training programs.^12^ Prehospital ALS is performed according to a protocol fixed by each municipality (ie, detailed protocols can vary among municipalities) based on the instructions of medical directors. In some municipalities, ALS by EMS personnel is restricted to patients aged over 8 years.

Critically ill and injured patients, including pediatric OHCA patients, are usually transported to a tertiary emergency medical centers, called emergency and critical care centers, which cover 500,000 people in each region. As of 2017, there were 300 (ie, 286 for all ages and 14 for children) emergency and critical care centers in Japan (Supplemental Table 1).^17^ Emergency and critical care centers are staffed with emergency and critical care physicians/surgeons, nurses, and other specialists, and operate 24 hours a day, 7 days a week for critically ill and injured patients. These centers have a similar level of capacity to perform sufficient treatment each other. Pediatric emergency and critical care centers are specialized for pediatric care.

**Figure 1:**
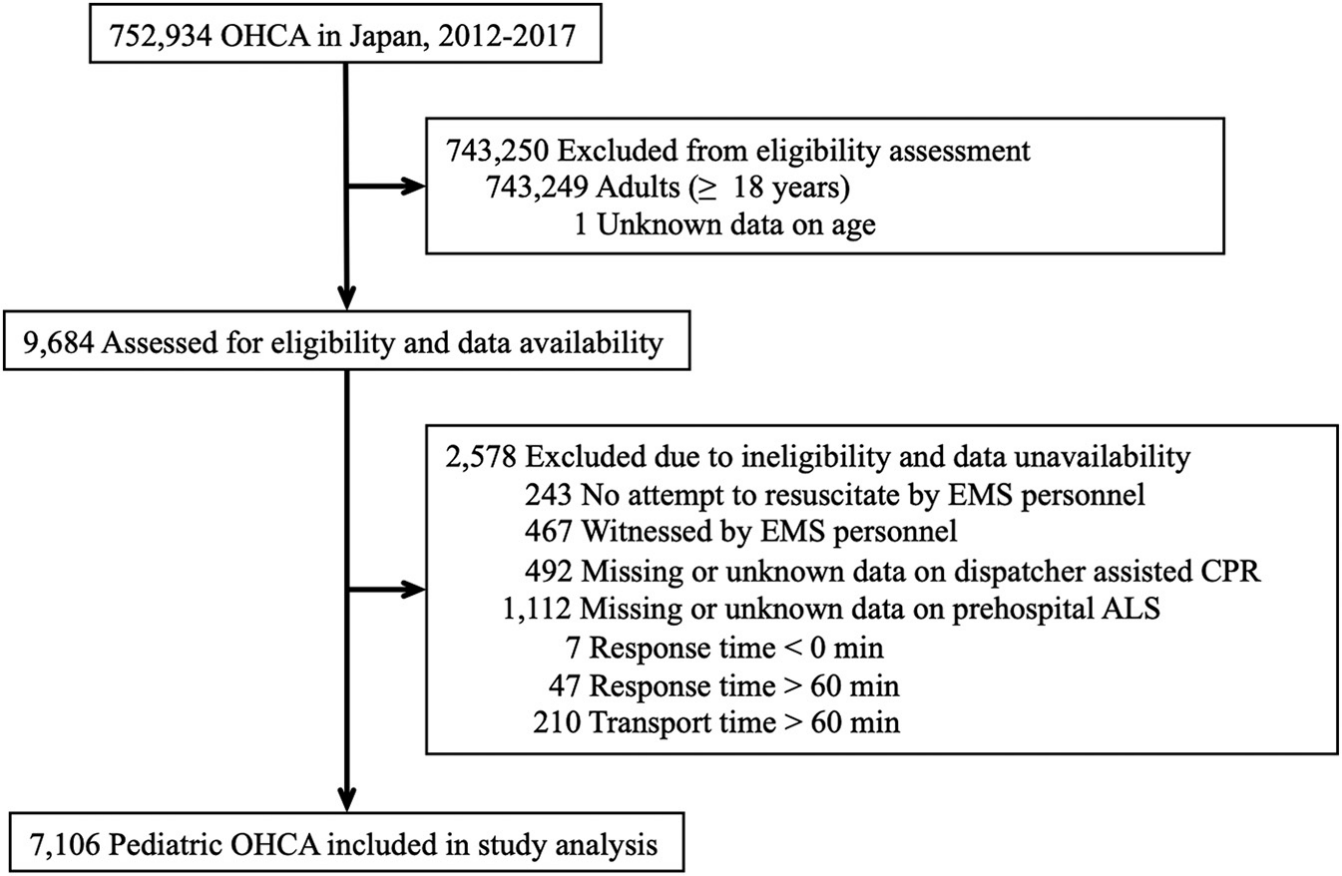
Patient Flow Diagram ALS = advanced life support; CPR = cardiopulmonary resuscitation; EMS = emergency medical service; OHCA = out-of-hospital cardiac arrest.

This study included pediatric (<18 years) OHCA patients submitted to the All-Japan Utstein Registry between January 1, 2012, and December 31, 2017. We excluded patients without CPR attempts by EMS personnel or patients with cardiac arrest witnessed by EMS personnel. In addition, we excluded patients with unrealistic or contradictory times (ie, response time <0 min), as well as patients who did not receive timely treatments (ie, response time >60 min or transport time >60 min), because in such cases, there may have been data entry errors or the patients may have been under extraordinary circumstances.^12^ We also excluded patients with missing, incomplete, or inconsistent data, which accounted for <1% of all OHCA patients (Figure 1).

The institutional review board of Toranomon Hospital and University of the Ryukyus approved this study with a waiver of informed consent due to the anonymous nature of the data. The study was conducted in accordance with the amended Declaration of Helsinki.

### Variables

Data on patient characteristics (ie, age and sex), bystander characteristics (ie, witness, bystander CPR, public-access defibrillation, and dispatcher-assisted CPR), cardiac arrest characteristics (ie, initial rhythm and etiology of arrest), event characteristics (ie, time and place of arrest; for seasons and regions, the classification defined by the Japan Meteorological Agency was used), and prehospital ALS characteristics (ie, physician involvement in prehospital ALS, intravenous line insertion, epinephrine administration, and advanced airway management) were collected. Data on a series of EMS activity times (ie, emergency call, contact with patient, and hospital arrival) were recorded by each EMS squad. Based on time variables recorded in whole minutes, response time was calculated as the time interval between emergency call and contact with patient, and transport time was calculated as the time interval between contact with patient and hospital arrival. In this study, day/evening was defined as 7:00 am to 22:59 pm and night as 23:00 pm to 6:59 am, similar to the definition used in previous studies.^22^^,^^23^ Weekday was defined as any day of the week, except Saturday and Sunday, and weekend was defined as Saturday and Sunday. Based on an inquiry for the receiving hospital, a 1-month follow-up survey was conducted by each fire department, and the etiology of the cardiac arrest was confirmed. At the same time, 1-month outcome data on survival and neurological status were also collected. If the patient was transferred or discharged from the hospital within 1 month of the event, further investigations were conducted by the fire department in cooperation with the hospital personnel.

### Outcomes

The primary outcome of our analysis was survival 1 month after OHCA. The secondary outcomes were prehospital return of spontaneous circulation and favorable neurological status 1 month after OHCA, defined as a Glasgow-Pittsburgh cerebral performance category score of 1 (good performance) or 2 (moderate disability).^18^^,^^24^

### Statistical analysis

Descriptive statistics were used to characterize the study cohort. Categorical variables were presented as counts with proportions, and differences between groups were evaluated using the chi-square test. Continuous variables were presented as the mean ± SD or median (IQR), and we evaluated differences between the groups using the Student *t* test or the Wilcoxon Mann-Whitney test.

To examine the independent association of both time of day (day/evening vs night) and day of week (weekday vs weekend) with outcomes after OHCA, multivariable logistic regression models were used. The following variables that could influence the outcomes after OHCA were included in the models: age, sex, witness (unwitnessed, witnessed by a family member, or witnessed by a non-family member), bystander CPR, public-access defibrillation, dispatcher’s instructions for CPR, initial rhythm (ventricular fibrillation, ventricular tachycardia, pulseless electrical activity, asystole, or others), etiology of arrest (cardiac, external, or noncardiac nonexternal cause), prehospital ALS (basic life support only, ALS by EMS personnel, or ALS by physician), response time, transport time, year of arrest, season of arrest (spring, summer, autumn, or winter), region of arrest (north, east, west, or south), time of day (day/evening or night), and day of the week (weekday or weekend). Adjusted odds ratios (ORs) with 95% CIs were calculated.

To further characterize the association between time of day (day/evening vs night) and 1-month survival after OHCA, we additionally conducted subgroup analyses according to several predefined subgroups: age (0 y, 1–7 y, or 8–17 y) and etiology of arrest (cardiac, external, or noncardiac nonexternal cause). Multivariable logistic regression models including the same set of variables used in the primary analyses were performed, and adjusted ORs of 1-month survival for patients during day/evening vs night were reported with 95% CIs.

As an ancillary analysis, a comparison between the current study and the previous study of pediatric OHCA in Japan was also conducted. In conformity with the previous study,^16^ we focused on pediatric patients with witnessed OHCA, and national holidays were regarded as weekends in these analyses.

Statistical analyses were conducted using JMP Pro 15.0.0 software (SAS Institute). A 2-sided *P* value of 0.05 was considered statistically significant.

## Results

During the study period, we identified 7,106 eligible pediatric patients with OHCA (Figure 1). The baseline characteristics are shown in Table 1. A total of 5,209 events (73.3%) occurred during day/evening hours, and 1,897 events (26.7%) occurred during night hours. A total of 5,010 events (70.5%) occurred on weekdays, and 2,096 events (29.5%) occurred on weekends. Overall, the mean age was 5.7 ± 6.5 years. Patients experiencing OHCA during the night were significantly younger than patients experiencing OHCA during the day/evening, and approximately one-half of the patients during the night (47.9%) were infants (<1 year). Compared with during day/evening, fewer patients had a witnessed event or received public-access defibrillation during the night, and the initial rhythm in most of the patients during the night (83.2%) was asystole. Patients at night had a lower chance of receiving ALS in the prehospital setting than patients in the day/evening. In the comparison between weekdays and weekends, there was almost no difference in the baseline characteristics. However, the response time was significantly longer on weekends than on weekdays.

**Table 1:** Baseline Characteristics According to Time of Day and Day of Week in the Full Cohort

	All Patients (N = 7,106)	Time of Day			Day of the Week		
		Day/Evening (n = 5,209)	Night (n = 1,897)	*P* Value	Weekday (n = 5,010)	Weekend (n = 2,096)	*P* Value
Age, y							
Mean ± SD	5.7 ± 6.5	6.1 ± 6.5	4.8 ± 6.4	<0.0001	5.9 ± 6.5	5.4 ± 6.3	0.0019
Median (IQR)	2 (0-13)	3 (0-13)	1 (0-11)	<0.0001	2 (0-13)	2 (0-12)	0.0079
Age group							
0 y	2,693 (37.9)	1,785 (34.3)	908 (47.9)	<0.0001	1,868 (37.3)	825 (39.4)	0.0019
≥1, <8 y	1,882 (26.5)	1,442 (27.7)	440 (23.2)		1,293 (25.8)	589 (28.1)	
≥8, <18 y	2,531 (35.6)	1,982 (38.0)	549 (28.9)		1,849 (36.9)	682 (32.5)	
Sex							
Male	4,328 (60.9)	3,218 (61.8)	1,110 (58.5)	0.0126	3,017 (60.2)	1,311 (62.5)	0.0666
Female	2,778 (39.1)	1,991 (38.2)	787 (41.5)		1,993 (39.8)	785 (37.5)	
Witness							
No witness	4,894 (68.9)	3,447 (66.2)	1,447 (76.3)	<0.0001	3,461 (69.1)	1,433 (68.4)	0.2053
By family member	1,319 (18.5)	979 (18.8)	340 (17.9)		906 (18.1)	413 (19.7)	
By non-family member	893 (12.6)	783 (15.0)	110 (5.8)		643 (12.8)	250 (11.9)	
Bystander CPR							
Yes	4,439 (62.5)	3,238 (62.2)	1,201 (63.3)	0.3763	3,131 (62.5)	1,308 (62.4)	0.9428
No	2,667 (37.5)	1,971 (37.8)	696 (36.7)		1,879 (37.5)	788 (37.6)	
Public-access defibrillation							
Yes	172 (2.4)	168 (3.2)	4 (0.2)	<0.0001	131 (2.6)	41 (2.0)	0.0994
No	6,934 (97.6)	5,041 (96.8)	1,893 (99.8)		4,879 (97.4)	2,055 (98.0)	
Dispatcher's instruction for CPR							
Yes	4,420 (62.2)	3,155 (60.6)	1,265 (66.7)	<0.0001	3,139 (62.7)	1,281 (61.1)	0.2226
No	2,686 (37.8)	2,054 (39.4)	632 (33.3)		1,871 (37.3)	815 (38.9)	
Initial rhythm							
VF	270 (3.8)	224 (4.3)	46 (2.4)	<0.0001	189 (3.8)	81 (3.9)	0.6134
VT	15 (0.2)	13 (0.2)	2 (0.1)		13 (0.2)	2 (0.1)	
PEA	1,005 (14.1)	805 (15.5)	200 (10.6)		720 (14.4)	285 (13.6)	
Asystole	5,235 (73.7)	3,657 (70.2)	1,578 (83.2)		3,679 (73.4)	1,556 (74.2)	
Others (eg, bradycardia)	581 (8.2)	510 (9.8)	71 (3.7)		409 (8.2)	172 (8.2)	
Etiology of arrest							
Cardiac cause	2,449 (34.5)	1,662 (31.9)	787 (41.5)	<0.0001	1,722 (34.4)	727 (34.7)	0.7275
External cause (eg, asphyxia, drowning, or trauma)	2,178 (30.6)	1,795 (34.5)	383 (20.2)		1,526 (30.4)	652 (31.1)	
Noncardiac nonexternal cause (eg, sepsis, respiratory disease, or malignancy)	2,479 (34.9)	1,752 (33.6)	727 (38.3)		1,762 (35.2)	717 (34.2)	
Prehospital ALS							
BLS only	5,014 (70.6)	3,595 (69.0)	1,419 (74.8)	<0.0001	3,498 (69.8)	1,516 (72.3)	0.0926
By EMS personnel	1,062 (14.9)	798 (15.3)	264 (13.9)		762 (15.2)	300 (14.3)	
By physician	1,030 (14.5)	816 (15.7)	214 (11.3)		750 (15.0)	280 (13.4)	
Response time, min							
Mean ± SD	9.1 ± 4.8	9.1 ± 5.0	9.2 ± 4.0	0.3407	9.0 ± 4.5	9.4 ± 5.4	0.0035
Median (IQR)	8 (7-10)	8 (7-10)	9 (7-10)	<0.0001	8 (7-10)	8 (7-10)	0.2878
Transport time, min							
Mean ± SD	21.9 ± 9.9	22.0 ± 10.0	21.6 ± 9.7	0.1244	22.0 ± 9.8	21.8 ± 10.1	0.4333
Median (IQR)	20 (15-27)	20 (15-27)	20 (15-26)	0.1335	20 (15-27)	20 (15-27)	0.1550
Year of arrest							
2012	1,538 (21.6)	1,139 (21.9)	399 (21.0)	0.2296	1,122 (22.4)	416 (19.9)	0.1340
2013	1,115 (15.7)	837 (16.1)	278 (14.7)		768 (15.3)	347 (16.6)	
2014	1,149 (16.2)	816 (15.6)	333 (17.5)		819 (16.4)	330 (15.7)	
2015	1,159 (16.3)	863 (16.6)	296 (15.6)		796 (15.9)	363 (17.3)	
2016	1,128 (15.9)	817 (15.7)	311 (16.4)		797 (15.9)	331 (15.8)	
2017	1,017 (14.3)	737 (14.1)	280 (14.8)		708 (14.1)	309 (14.7)	
Season of arrest							
Spring (March, April, May)	1,862 (26.2)	1,339 (25.7)	523 (27.6)	0.0018	1,322 (26.4)	540 (25.8)	0.3494
Summer (June, July, August)	1,720 (24.2)	1,312 (25.2)	408 (21.5)		1,204 (24.0)	516 (24.6)	
Autumn (September, October, November)	1,585 (22.3)	1,178 (22.6)	407 (21.4)		1,140 (22.8)	445 (21.2)	
Winter (December, January, February)	1,939 (27.3)	1,380 (26.5)	559 (29.5)		1,344 (26.8)	595 (28.4)	
Region of arrest							
North	689 (9.7)	503 (9.6)	186 (9.8)	0.5729	494 (9.9)	195 (9.3)	0.4809
East	3,847 (54.1)	2,827 (54.3)	1,020 (53.8)		2,717 (54.2)	1130 (53.9)	
West	2,449 (34.5)	1,797 (34.5)	652 (34.4)		1,708 (34.1)	741 (35.4)	
South	121 (1.7)	82 (1.6)	39 (2.0)		91 (1.8)	30 (1.4)	

Values are mean ± SD, median (IQR), or n (%), unless otherwise indicated.

ALS = advanced life support; BLS = basic life support; CPR = cardiopulmonary resuscitation; EMS = emergency medical service; PEA = pulseless electrical activity; VF = ventricular fibrillation; VT = ventricular tachycardia.

**Figure 2:**
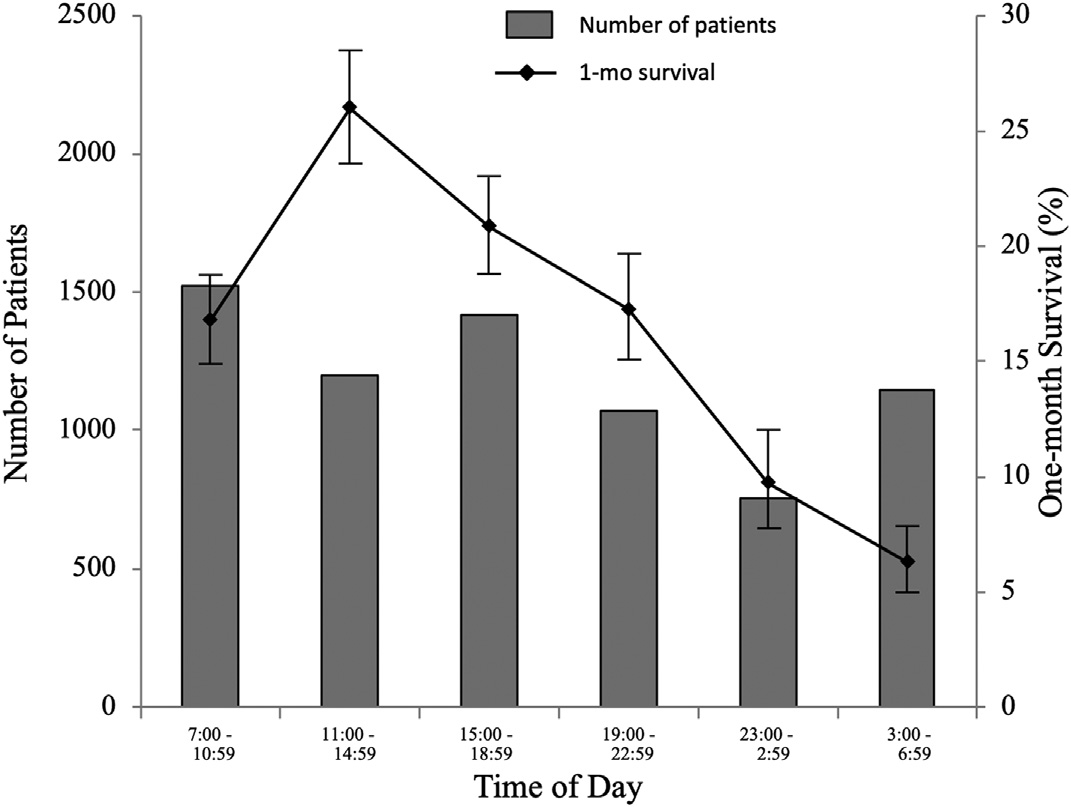
Number of Patients and Rate of 1-Month Survival by Time of Day Time of day was categorized into 6 periods of time according to 4-hour intervals. One-month survival was highest during 11:00 am to 14:59 pm and lowest during 3:00 am to 6:59 am (*P* < 0.0001).

**Figure 3:**
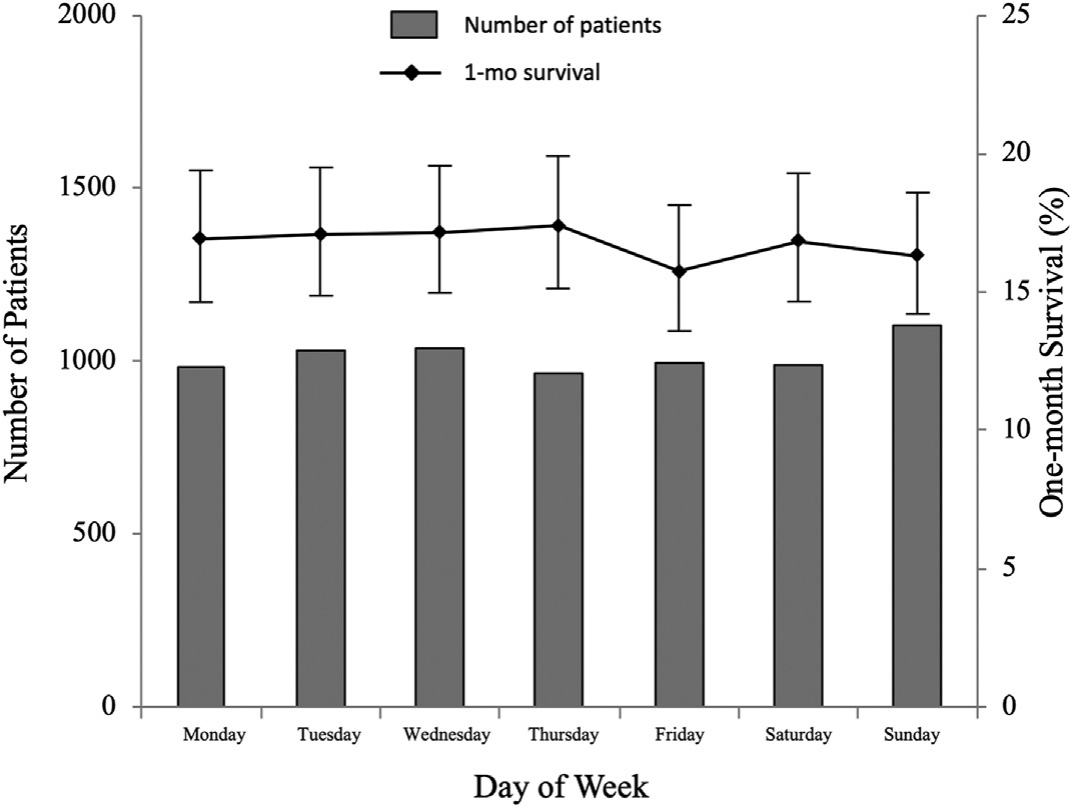
Number of Patients and Rate of 1-Month Survival by Day of the Week No difference was observed in 1-month survival between the days of the week (*P* = 0.9672).

Of the 7,106 pediatric OHCA patients, 1,192 (16.8%) survived 1 month after OHCA. Unadjusted 1-month survival was characterized by the time of day (categorized into 6 periods of time according to 4-hour intervals) in Figure 2 and by the day of the week in Figure 3. One-month survival was highest during 11:00 am to 14:59 pm and lowest during 3:00 am to 6:59 am (*P* < 0.0001). No difference was observed in 1-month survival between the days of the week (*P* = 0.9672).

Tables 2 and 3 present the outcomes of pediatric OHCA patients according to time of day (day/evening vs night) and day of week (weekday vs weekend), respectively. After adjusting for potential confounders, 1-month survival during the day/evening (1,047/5,209 [20.1%]) was significantly higher than that at night (145/1,897 [7.6%]) (adjusted OR: 2.31 [95% CI: 1.87–2.86]), whereas there was no significant difference in 1-month survival between weekdays (845/5,010 [16.9%]) and weekends (347/2,096 [16.6%]) (adjusted OR: 1.04 [95% CI: 0.88–1.23]) (Central Illustration). Similar associations were observed for prehospital return of spontaneous circulation and 1-month neurologically favorable survival.

**Table 2:** Outcomes for Pediatric OHCA During Day/Evening vs Night

	Day/Evening (n = 5,209)	Night (n = 1,897)	Adjusted OR (95% CI)	*P* Value
Primary outcome				
1-month survival	1,047 (20.1)	145 (7.6)	2.31 (1.87-2.86)	<0.0001
Secondary outcomes				
Prehospital ROSC	758 (14.6)	96 (5.1)	1.97 (1.48-2.62)	<0.0001
Favorable neurological outcome, CPC 1 or 2^a^	547 (10.6)	59 (3.1)	2.01 (1.40-2.87)	0.0001
CPC 1	503 (9.7)	54 (2.8)	NA	NA
CPC 2	44 (0.8)	5 (0.3)	NA	NA
CPC 3	149 (2.9)	21 (1.1)	NA	NA
CPC 4	309 (5.9)	58 (3.1)	NA	NA
CPC 5	4,156 (79.8)	1,750 (92.2)	NA	NA
CPC unknown	48 (0.9)	9 (0.5)	NA	NA

Values are n (%), unless otherwise indicated.

The association between time of day (day/evening vs night) and 1-month survival after pediatric OHCA was reported as adjusted ORs with 95% CIs.

CPC = cerebral performance category; NA = not applicable; OHCA = out-of-hospital cardiac arrest; OR = odds ratio; ROSC = return of spontaneous circulation.

a 57 patients for whom 1-month neurological status was not available were excluded from analysis

**Table 3:** Outcomes for Pediatric OHCA During Weekday vs Weekend

	Weekday (n = 5,010)	Weekend (n = 2,096)	Adjusted OR (95% CI)	*P* Value
Primary outcome				
1-month survival	845 (16.9)	347 (16.6)	1.04 (0.88-1.23)	0.6671
Secondary outcomes				
Prehospital ROSC	608 (12.1)	246 (11.7)	1.01 (0.81-1.27)	0.8990
Favorable neurological outcome, CPC 1 or 2^a^	424 (8.5)	182 (8.8)	0.89 (0.68-1.15)	0.3688
CPC 1	388 (7.8)	169 (8.1)	NA	NA
CPC 2	36 (0.7)	13 (0.6)	NA	NA
CPC 3	124 (2.5)	46 (2.2)	NA	NA
CPC 4	261 (5.2)	106 (5.0)	NA	NA
CPC 5	4,160 (83.0)	1,746 (83.3)	NA	NA
CPC unknown	41 (0.8)	16 (0.8)	NA	NA

Values are n (%), unless otherwise indicated.

The association between day of week (weekday vs weekend) and 1-month survival after pediatric OHCA was reported as adjusted ORs with 95% CIs.

Abbreviations as in Table 2.

a 57 patients for whom 1-month neurological status was not available were excluded from analysis.

**Central Illustration:**
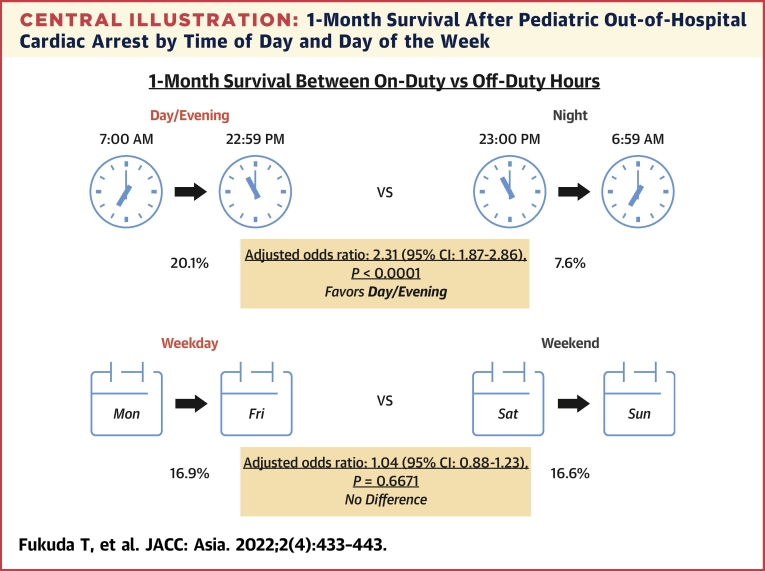
1-Month Survival After Pediatric Out-of-Hospital Cardiac Arrest by Time of Day and Day of the Week This study examined whether the rate of survival after pediatric out-of-hospital cardiac arrest was affected by the time of day or day of the week when the event occurred. The rate of 1-month survival was lower at night than during the day/evening, whereas there was no difference in 1-month survival between weekday and weekend.

**Figure 4:**
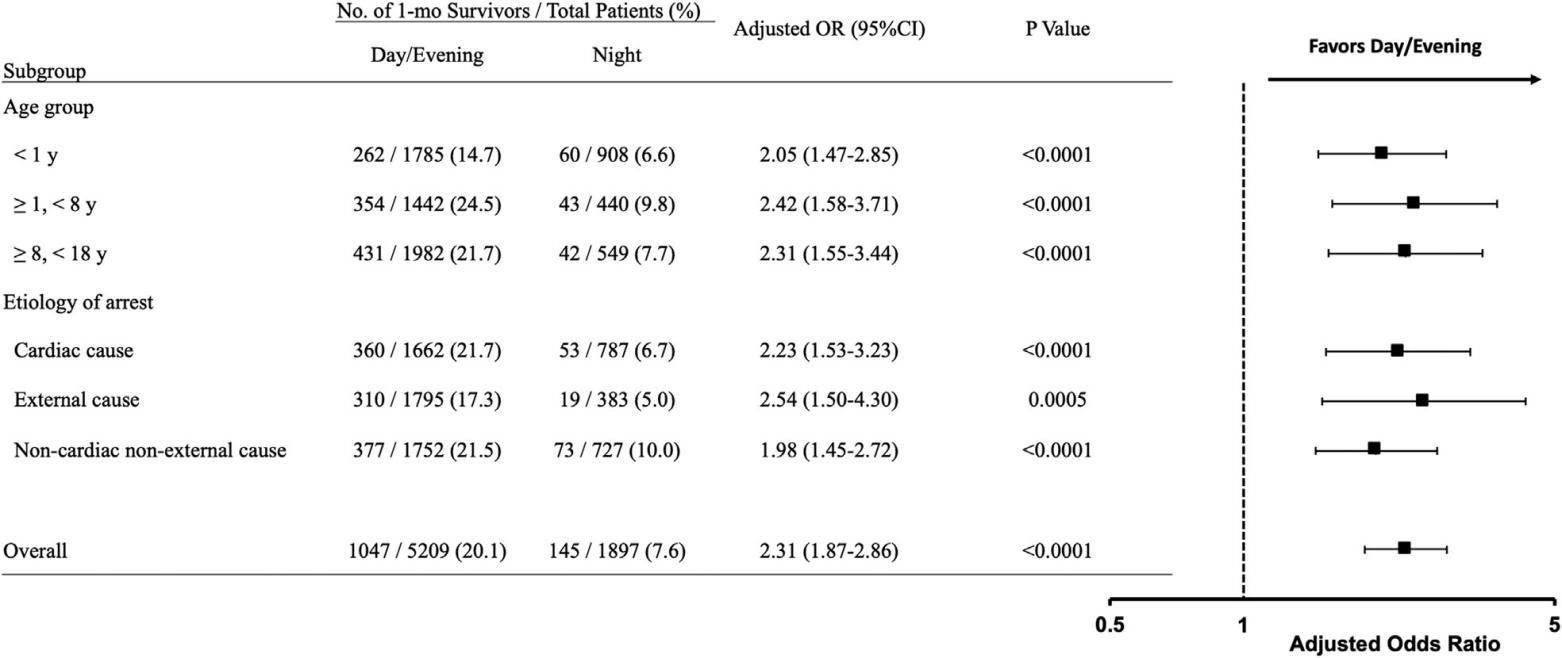
Adjusted ORs of 1-Month Survival for the Prespecified Subgroups The associations between time of day (day/evening vs night) and 1-month survival after pediatric out-of-hospital cardiac arrest were reported as adjusted ORs with 95% CIs for the prespecified subgroups according to age (0 years, 1–7 years, or 8–17 years) and etiology of arrest (cardiac, external, or noncardiac nonexternal cause). Multivariable logistic regression models were performed by including the same set of variables used in the primary analyses.

In the subgroup analyses (Figure 4), the association between time of day (day/evening vs night) and 1-month survival did not change regardless of age or etiology of arrest.

In the ancillary analysis (Supplemental Table 2), similar to the findings of our primary analysis, there was a significant difference between day/evening and night, but not between weekdays and weekends/holidays. Over time (Supplemental Figure 1), 1-month survival tended to improve regardless of the time of day and day of the week among pediatric patients with witnessed OHCA. Disparities in unadjusted survival between day/evening and night remained, but those between weekdays and weekends/holidays narrowed.

## Discussion

In this nationwide population-based observational study of pediatric OHCA, the rate of 1-month survival was lower at night compared with day/evening, whereas there was no difference in 1-month survival between weekdays and weekends, after adjusting for potential confounders. The observational study design precludes ascertainment of causality. However, the large sample size based on a government-led nationwide population-based registry that routinely collected data for all OHCA patients transported to an emergency hospital and the consistency in multiple analyses ensured the statistical robustness of our findings.

The findings of our study (treating 7,106 pediatric OHCA patients in Japan from 2012 to 2017) were inconsistent with the findings of a previous study (treating 3,278 pediatric OHCA patients in Japan from 2005 to 2011).^16^ The previous study found that pediatric OHCA occurring during both night (adjusted OR: 0.68 [95% CI: 0.56–0.82]) and weekend (adjusted OR: 0.79 [95% CI: 0.65–0.97]) were significantly associated with a decreased chance of 1-month survival.^16^ Subsequently, we found that there was no difference in 1-month survival after pediatric OHCA between on weekdays and weekends, although 1-month survival remained significantly lower during the night than during the day/evening. This partial improvement may be partly due to the Japanese government’s policy efforts. In Japan, in addition to the subsidy for maintaining and enhancing the EMS system, the government has provided subsidies of several million dollars every year since 2010 for the operation of pediatric emergency and critical care centers. The objectives of this project are to develop designated pediatric emergency and critical care centers: 1) to accept critically ill and injured pediatric patients 24 hours a day, 7 days a week; 2) to provide specialized care and intensive care following hyperacute care; and 3) to develop human resources involved in pediatric emergency care. Owing to this project, the number of emergency and critical care centers, as well as health care professionals, who provide pediatric emergency care has continued to increase steadily (Supplemental Table 1).^12^^,^^17^ Considering that the survival disparity between day/evening and night was larger than that between weekdays and weekends in the previous study,^16^ our findings may imply that it is easier to correct the disparities between weekdays and weekends than between day/evening and night.

However, it is uncertain whether promoting the establishment of more emergency and critical care centers can reduce the survival disparities between day/evening and night. If we expand the scope of the search from pediatric OHCA to include adult OHCA in Japan, a previous study showed that the rate of survival from adult OHCA was higher during day/evening than at night (adjusted OR: 1.26 [95% CI: 1.22–1.31]), but did not differ between weekends/holidays and weekdays (adjusted OR: 1.00 [95% CI: 0.96–1.04]).^25^ The survival disparity between day/evening and night was observed even in mature adult EMS system in Japan. If we expand the scope of the search from OHCA to include in-hospital cardiac arrest (IHCA), a larger study (treating 12,404 pediatric IHCA patients in North America from 2000 to 2012) can be identified.^26^ That study found that the rate of survival from pediatric IHCA was lower during the night than during day/evening (adjusted OR: 0.88 [95% CI: 0.80–0.97]), but did not differ between weekends and weekdays (adjusted OR: 0.92 [95% CI: 0.84–1.01]). These findings were consistent with the findings of our study, regardless of the difference in the settings, including country as well as the EMS system or the hospital system for pediatric resuscitation. There might be common reasons why night was associated with a decreased chance of survival after pediatric cardiac arrest (eg, physiological basis). Alternatively, residual unmeasured confounders may have made an apparent difference between day/evening and night. The possible unmeasured confounders were as follows: a night shift can affect health care professionals’ cognitive and psychomotor performance and may increase clinically significant medical errors.^27^^,^^28^ Staffing of hospitals, especially the number of senior health care professionals, is typically reduced during the night, although the intensity or level of staffing seems to be associated with hospital mortality.^29^, ^30^, ^31^ During the night, the availability of resources and personnel is restricted, although pediatric readiness is essential for pediatric resuscitation requiring advanced and specialized care.^32^ In our study, we could not adjust for such unmeasured confounders. It is important to identify potential causes for the decreased survival after pediatric cardiac arrest during the night to determine whether some measures can be taken against the causes. This warrants further investigation.

Even if increasing the number of facilities and health care professionals for pediatric resuscitation is effective in improving survival after pediatric OHCA, there is a limit to this plan due to limited human resources. Over the past few decades, the Japanese government has promoted medical care plans to increase the number of emergency and critical care centers that can provide advanced and highly specialized care for critically ill and injured pediatric patients 24 hours a day, 365 days a year with the aim of enhancing the pediatric emergency care system. On the other hand, in recent years, the Japanese government has also promoted work style reform that includes the regulation of long working hours for physicians.^33^, ^34^, ^35^, ^36^ To resolve these contradictory issues, high expectations are placed on task shifting and task sharing.^37^^,^^38^ Again, to practice strategic staffing, potential causes for decreased survival after pediatric cardiac arrest during the night should be identified in detail. Moreover, further studies are required to determine whether the survival disparities between day/evening and night can be eliminated by strategic staffing.

### Study limitations

Several limitations should be considered when interpreting the findings of our study. First, the observational study design could only derive association rather than causality, as the possibility of residual selection bias and unmeasured confounders remain. Second, the generalizability of our findings to other countries is uncertain. The population of pediatric OHCA patients may vary by country. In addition, different EMS systems or hospital staffing patterns during the night could lead to different results. Third, we could not obtain information on hospitals where patients were transported to from the All-Japan Utstein Registry database. Although each emergency and critical care center operates 24 hours a day, 7 days a week and has a similar level of capability, the lack of information on those hospitals could potentially affect the results of our analyses. Fourth, data on in-hospital or post-resuscitation care were not available from the All-Japan Utstein Registry database. Although the All-Japan Utstein Registry data, based on the Utstein templates, allow for the adjustment for a large number of potential confounders, unmeasured confounders including inpatient data cannot be adjusted for. Fifth, our study was not designed to identify the underlying causes of survival disparities between on-duty hours and off-duty hours, although our study highlights an important public health issue. Further research is required to determine the mechanisms that cause survival disparities between day/evening and night, which may have important implications for hospital staffing, shift scheduling, training, or resource allocation. Finally, our study lacks an economic perspective. A formal cost-effectiveness study would be required before putting the plan into practice, even if further research reveals that appropriate hospital staffing, resource allocation, or education can eliminate survival disparities between day/evening and night.

## Conclusions

We found that the rate of 1-month survival after pediatric OHCA was lower at night compared with day/evening, whereas there was no difference between weekdays and weekends, after adjusting for potential confounders. Although there are several plausible mechanisms that may cause survival disparities between day/evening and night, further research should explore the underlying causes of survival disparities. A clearer understanding of the reasons for these disparities will play an important role in policy making to achieve parity in treatment outcomes between on-duty hours and off-duty hours among critically ill and injured pediatric patients.

Perspectives **COMPETENCY IN MEDICAL KNOWLEDGE:** This study showed that 1-month survival rates of pediatric OHCA in Japan between 2012 and 2017 were significantly lower at night compared with day/evening, whereas there was no difference in 1-month survival between weekdays and weekends. Compared with the past, disparities in 1-month survival between weekdays and weekends have been eliminated, although those between day/evening and night remain.

**TRANSLATIONAL OUTLOOK:** Further research should explore the underlying causes of survival disparities between day/evening and night. A clearer understanding of the reasons for these disparities will play an important role in policy making to achieve parity in treatment outcomes between on-duty hours and off-duty hours among critically ill and injured pediatric patients.

## Funding Support and Author Disclosures

This work was supported by the Japan Foundation for Pediatric Research Grant No. 20-014 and JSPS KAKENHI Grant No. JP21K17252 (Dr Fukuda). The authors have reported that they have no relationships relevant to the contents of this paper to disclose.
