# Supplementary material for: Survival From Pediatric Out-of-Hospital Cardiac Arrest During Nights and Weekends: An Updated Japanese Registry-Based Study
Source: JACC Asia. 2022 May 10;2(4):433–43. doi: 10.1016/j.jacasi.2022.01.005 (PMC9627930; doi:10.1016/j.jacasi.2022.01.005)
Supplement: Supplemental Figure 1 and Tables 1 and 2 [file mmc1.docx]

**SUPPLEMENTAL MATERIAL
Survival From Pediatric Out-of-Hospital Cardiac Arrest During Nights and Weekends: an Updated Japanese Registry-Based Study**

**Running title:** Night and weekend effect among pediatric OHCA

Tatsuma Fukuda, MD, PhD^1,2*^, Naoko Ohashi-Fukuda, MD^3^, Hiroshi Sekiguchi, RN, MMSc, PhD^2^, Ryota Inokuchi, MD, PhD^4^, Ichiro Kukita, MD, PhD^2^

**Supplementary Appendix**

This appendix has been provided by the authors to give readers additional information about their work.

**Table of Contents**

**Supplemental Table 1.** Change in the number of emergency and critical care centers in Japan, 2005–2017……………………………………………………………………………2

**Supplemental Table 2.** Adjusted odds ratios of one-month survival in the previous study (2005–2011) and the current study (2012–2017)…………………………………………… 3

**Supplemental Figure 1.** Temporal trends and disparities in one-month survival by time of day and day of week among pediatric patients with witnessed OHCA………………………4

**Supplemental Table 1.** Change in the number of emergency and critical care centers in Japan, 2005–2017

|  | 2005 | 2006 | 2007 | 2008 | 2009 | 2010 | 2011 | 2012 | 2013 | 2014 | 2015 | 2016 | 2017 |
| --- | --- | --- | --- | --- | --- | --- | --- | --- | --- | --- | --- | --- | --- |
| Emergency and Critical Care Center | 178 | 189 | 201 | 208 | 214 | 221 | 235 | 249 | 259 | 266 | 271 | 279 | 286 |
| Pediatric Emergency and Critical Care Center | 1 | 1 | 2 | 2 | 2 | 4 | 4 | 4 | 6 | 8 | 8 | 11 | 14 |

In Japan, emergency and critical care centers are staffed with emergency and critical care physicians/surgeons, nurses, and other specialists and operate 24 hours a day, 7 days a week for critically ill and injured patients. A pediatric emergency and critical care center specializes in pediatric care. Over the past few decades, the Japanese government has promoted medical care plans to increase the number of adult and pediatric emergency and critical care centers.

**Supplemental Table 2.** Adjusted odds ratios of one-month survival in the previous study (2005–2011) and the current study (2012–2017)

|  | 2005–2011 Kitamura 2014 | | 2012–2017 Fukuda 2021* | |
| --- | --- | --- | --- | --- |
|  | No. of 1-mo Survivors / Witnessed Patients (%) | Adjusted OR (95%CI) | No. of 1-mo Survivors / Witnessed Patients (%) | Adjusted OR (95%CI) |
| Night  vs Day/Evening** | 289/1870 (15.5%)  vs 328/1408 (23.3%) | 0.68 (0.56-0.82) | 96/450 (21.3%)  vs 625/1762 (35.5%) | 0.54 (0.40-0.73) |
| Weekend/Holiday  vs Weekday*** | 175/1114 (15.7%)  vs 442/2164 (20.4%) | 0.79 (0.65-0.97) | 230/743 (31.0%)  vs 491/1469 (33.4%) | 0.99 (0.78-1.25) |

These analyses were performed by focusing on pediatric patients with witnessed OHCA.

* In the multivariable logistic regression model, the same set of variables used in the primary analyses were included.

** In the current study, day/evening was defined as 7:00 AM to 22:59 PM, and night as 23:00 PM to 6:59 AM. In the previous study, daytime was defined as 9:00 AM to 16:59 PM, and nighttime as 17:00 PM to 8:59 AM.

*** In these analyses, weekday was defined as any day of the week except Saturday and Sunday, and weekend was defined as Saturday and Sunday. In addition, national holidays were regarded as weekends.

Abbreviations: OHCA, Out-of-hospital cardiac arrest; OR, Odds ratio

**Supplemental Figure 1. Temporal trends and disparities in one-month survival by time of day and day of week among pediatric patients with witnessed OHCA**

~~~~

In the current study, day/evening was defined as 7:00 AM to 22:59 PM and night as 23:00 PM to 6:59 AM. In the previous study, daytime was defined as 9:00 AM to 16:59 PM, and nighttime as 17:00 PM to 8:59 AM.

In this analysis, national holiday was regarded as weekend.

Abbreviations: OHCA, Out-of-hospital cardiac arrest
